# Intellectual Disabilities and Neurocognitive Impairment in Adult Patients with Inherited Metabolic Diseases: A UK Single Centre Experience

**DOI:** 10.3390/genes15070923

**Published:** 2024-07-15

**Authors:** John Warner-Levy, Adrian H. Heald, Daniel Hand, Reena Sharma, Rachel Thomasson, Karolina M. Stepien

**Affiliations:** 1 Department of Diabetes and Endocrinology, Salford Royal Organisation, Northern Care Alliance NHS Foundation Trust, Salford M6 8HD, UK; jwarnerlevy@gmail.com (J.W.-L.); adrian.heald@nca.nhs.uk (A.H.H.); 2 The School of Medicine and Manchester Academic Health Sciences Centre, The University of Manchester, Manchester M13 9PL, UK; 3 Adult Inherited Metabolic Disorders, Salford Royal Organisation, Northern Care Alliance NHS Foundation Trust, Salford M6 8HD, UK; daniel.hand@nca.nhs.uk (D.H.); reena.sharma@nca.nhs.uk (R.S.); 4 Neurology and Neuropsychiatry Department, Salford Royal Organisation, Northern Care Alliance NHS Foundation Trust, Salford M6 8HD, UK; rachel.thomasson@nca.nhs.uk; 5 The Division of Cardiovascular Sciences, The University of Manchester, Manchester M13 9PL, UK

**Keywords:** intellectual disability, neurocognitive dysfunction, inherited metabolic diseases, rare disease, neurocognitive assessment

## Abstract

Inherited metabolic diseases (IMDs) are a group of heterogeneous genetic disorders resulting in substrate accumulation, energy deficiency, or complex molecular defects due to the failure of specific molecules to act as enzymes, cofactors, transporters, or receptors in specific metabolic pathways. The pathophysiological changes seen in IMDs are sometimes associated with intellectual disability (ID) or neurocognitive decline, necessitating multidisciplinary input. We here describe our experience at one tertiary metabolic centre in the UK. We reviewed the case prevalence and existing service provision in one adult IMD service covering a multi-ethnic population of 10 million in North England. In our cohort of 2268 IMD patients, 1598 patients had general metabolic conditions (70.5%), and 670 had lysosomal storage disease/disorders (LSD)s (29.5%). The overall prevalence of ID and neurocognitive decline was found to be 15.7% (*n* = 357), with patients with LSDs accounting for 23.5% (*n* = 84) of affected patients. Given the prevalence of ID in adults with IMDs, access to multidisciplinary input from neuropsychology and neuropsychiatry services is important. Education of healthcare professionals to diagnose IMDs in patients with ID, in addition to neurocognitive and neuropsychiatric presentations, will avoid missed diagnoses of IMD and will have a positive effect on patient outcomes.

## 1. Introduction

Inherited metabolic diseases (IMDs) encompass a range of heterogeneous genetic disorders characterised by the accumulation of substrates, various deficiencies, or complex molecular defects, with the majority exhibiting a recessive mode of inheritance [1]. In general, IMDs fall into one of three categories: mechanism-intoxication type, energy deficit type, and disorders affecting the degradation of complex molecules leading to storage [2]. As this usually occurs secondary to the failure of specific molecules to act as enzymes, cofactors, receptors or transporters in metabolic pathways, the pathophysiological changes seen in IMDs occur on a subcellular level and therefore may manifest systemically as opposed to affecting individual organs [3]. The combined occurrence of different IMDs leads to a noteworthy prevalence of 1 in 784 live births, which is significantly higher than previously suggested [4]. Although it was reported in 2022 that approximately 20,000 children and adults in the UK are living with an IMD [5], the number of adult patients with an IMD is increasing due to newborn screening and improved treatments in childhood, adolescence, and adulthood, leading to increased survival rates [6].

The Department of Health and Social Care define ID as ‘*a significantly reduced ability to understand new or complex information, to learn new skills (impaired intelligence), with a reduced ability to cope independently (impaired social functioning), which started before adulthood*’ [7]. An ID is generally static and apparent early in the development of an individual. Although neurocognitive impairment is a feature of ID, it can present independently in adulthood and may be a dynamic process, often as a decline.

In the UK, it has been reported that between 230,000 and 350,000 people are living with a severe intellectual disability (ID), and between 580,000 and 1,750,000 are living with a mild ID [8]. The prevalence of ID has not been clearly defined in adults with an IMD and an unknown number of patients with unrecognised adult-onset IMDs may be attending one or a combination of psychiatry, ID, and neurology services [9]. The Newborn Blood-Spot Test screens for six of the most common IMDs [10], but beyond this, a great degree of clinical suspicion is required from clinicians to diagnose an IMD. Although NHS Scotland and certain NHS trusts in England have guided clinicians on when to suspect and how to screen for IMDs [11,12], many clinicians remain uninformed and require further educational resources. In Canada, an app has been developed to aid specialists in evaluating children with a potential IMD [13].

The wide application of next-generation genomic and metabolomic testing [14,15,16] in clinical practice has enhanced the diagnostic pathway for many patients with ID [13]. The increasing knowledge about the genetic basis of disease and better understanding of the disease pathophysiology led to advances in therapeutic developments, e.g., gene therapy or haematopoietic stem cell therapy. The new therapeutic modalities target the central nervous system and therefore are powerful strategies for many neurodegenerative conditions including many IMDs.

As the delayed diagnosis of an IMD can lead to worsened patient outcomes and an increased NHS resource expenditure [17], we investigated the prevalence of ID and neurocognitive decline among adults with IMDs using data from our supra-regional clinic in the North of England. We aim to share the experience of a UK adult IMD service, also outlining available interventions and the multidisciplinary efforts needed for sustained long-term symptom management.

## 2. Materials and Methods

### 2.1. Study Design

This is a retrospective analysis of the data collected as part of routine care. The project is a health improvement project.

### 2.2. Population

We reviewed the existing service provision in one adult IMD service covering a multi-ethnic population of 10 million in North England. This Metabolic Centre looks after 2271 adult patients with inherited metabolic disorders (IMDs). The integrated hospital and primary care digital care record for Greater Manchester (GMCR) (https://gmwearebettertogether.com, accessed on 20 January 2024) and National Summary Care Record (https://digital.nhs.uk/services/national-care-records-service, accessed on 20 January 2024) were both used to access the primary care diagnoses, health issues, and prescribed medication of (1) patients with IMDs diagnosed in childhood who remain under the Adult Metabolic Service, and (2) adult patients who presented with progressive neurocognitive dysfunction in their adulthood before the diagnosis of an IMD was made.

### 2.3. Methods

The metabolic patient database was reviewed individually for evidence of ID through formal neuropsychological assessment undertaken in childhood. In cases where no neuropsychological assessment was undertaken, historical evidence of ID was proactively reviewed by experienced clinicians and/or neuropsychiatrist. This was stratified according to the Department of Health and Social definitions [7]. The search terms used were: ‘intellectual disability’, ‘learning disability’, ‘neurocognitive decline’, ‘mental health problems’, ‘psychosis’, ‘behavioural problems’, ‘carer support’, ‘social health support’, ‘neuropsychologist’, and ‘neuropsychiatrist’. The severity of ID is classified as mild, moderate, severe, or profound [18,19], as follows:

Mild—approximate IQ range of 50 to 69. Likely to result in some difficulties in the acquisition and comprehension of complex language concepts and academic skills. Most people can manage basic self-care, domestic, and practical activities, and can live and work relatively independently, but may require appropriate support.

Moderate—approximate IQ range of 35 to 49. Likely to have basic language and academic skills, but some will manage basic self-care, domestic, and practical activities. Most will need considerable and consistent support to live and work independently.

Severe—approximate IQ range of 20 to 34. Have very limited language and academic skills and may also have motor impairments. Typically need daily support in a supervised environment for adequate care but may acquire basic self-care skills with intensive training.

Profound—IQ under 20. Results in very limited communication skills and may have basic concrete skills. May have motor and sensory impairments, and typically need daily support in a supervised environment for adequate care.

For this analysis, we defined an ID term as a childhood onset, and neurocognitive decline (CI) as an adult-onset cognitive impairment, which is a dynamic phenomenon that progresses.

## 3. Results

### 3.1. Prevalence of ID/Neurocognitive Decline

**Figure 1:**
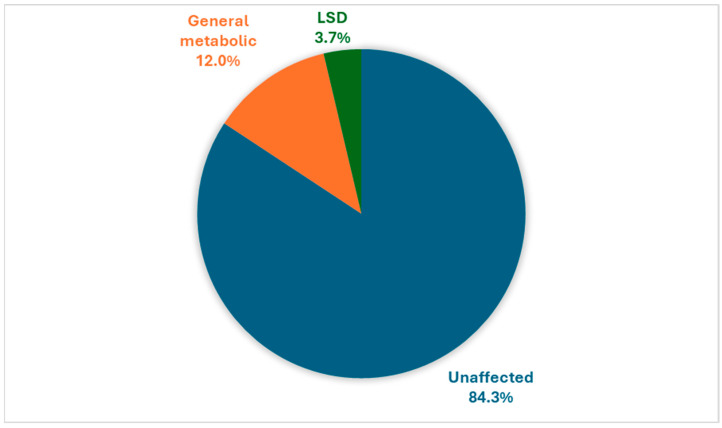
Illustration of the distribution of ID among 2268 IMD patients. The majority of patients, 84.3%, exhibit no ID. A smaller proportion, 12.0%, have ID in the context of general metabolic disorders. An even smaller group, 3.7%, displayed ID within the cohort diagnosed with LSDs.

In this adult clinic population of 2268 patients, 1598 patients had general metabolic conditions (70.5%), and 670 had LSDs (29.5%). The pooled prevalence of ID and neurocognitive decline was found to be 15.7% (*n* = 357), with patients with general metabolic conditions and LSDs accounting for 76.5% (*n* = 273) and 23.5% (*n* = 84) of affected patients, respectively, as seen in Figure 1.

**Figure 2:**
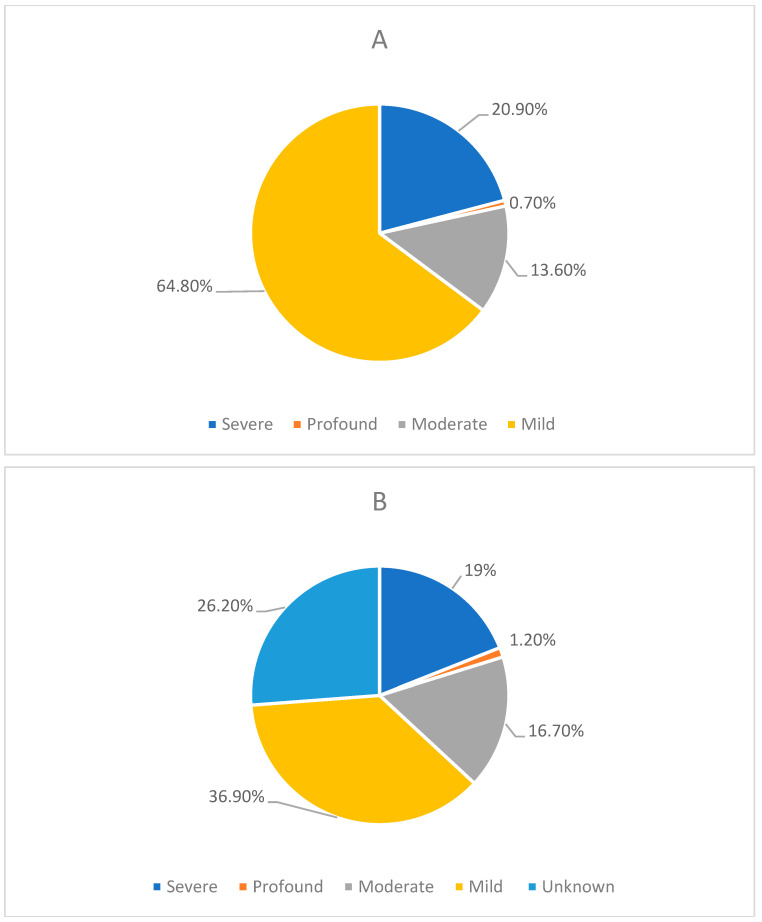
The severity of ID in patients with A. general metabolic conditions and B. LSDs. (**A**) The distribution of ID severity among individuals diagnosed with general metabolic disorders. The majority of patients experience mild ID (64.8%), followed by those with severe ID (20.9%). A smaller portion of patients have moderate ID (13.6%), while only a few individuals exhibit profound ID/CI (0.7%). (**B**) The distribution of ID severity among individuals diagnosed with LSDs. The severities are categorised as follows: Mild (36.9%), Unknown (26.2%), Severe (19.0%), Moderate (16.7%), and Profound (1.2%).

Regarding the severity of ID for patients with general metabolic disorders and LSDs, 64.8% (*n* = 177) and 36.9% (*n* = 31) were classified as mild, respectively. Figure 2 displays a detailed breakdown of ID severities.

The majority of these patients presented with ID in childhood (*n* = 317 out of 357), and sub-sequent investigations led to a diagnosis of an IMD. In some cases, ID was part of the syndrome they were affected with. It was not possible to establish whether ID was the first presenting symptom in these patients, as the data from the paediatric hospital were archived after the transfer of care to adult services. In some cases of childhood-onset PKU, classical galactosemia, arginase deficiency, SSADH, OTC deficiency, Smith–Lemli–Opitz syndrome, CTX, MTHFR, GAMT deficiency, Tango syndrome, MPS I, Mucolipidosis IV, MPS IIIA, β Mannosidosis, MPS VII, and Schindler’s syndrome, the diagnosis was not made until later in life (between 20–70 years) (*n* = 21). As a result, some of these patients were institutionalized for many years before the diagnosis of an IMD was made (PKU, classical galactosemia).

A small proportion of the IMD patients with ID (*n* = 40 out of 357) presented with neuro-cognitive decline in adulthood and the neuropsychiatry or neurology evaluation in consultation with our metabolic team led to a diagnosis of an IMD.

### 3.2. Prevalence of Specific IMDs within the Cohort

The most prevalent conditions in patients with ID for general metabolic disorders are described in Table 1, and all conditions in patients with ID for LSDs are described in Table 2. The most commonly defined general metabolic disorders associated with ID were phenylketonuria (PKU), classic galactosaemia, glucose transporter type 1 (GLUT-1) deficiency, arginase deficiency, and homocystinuria. The most common LSDs associated with ID and neurocognitive decline were Niemann–Pick C disease, Mucopolysaccharidosis type I (MPS I), α-mannosidosis, Mucopolysaccharidosis type II (MPS II), and Mucopolysaccharidosis type III (MPS III).

### 3.3. Referral Patterns

Between 2018 and 2023, we observed a twofold increase in referrals of adult metabolic patients (from 16 to 33 per year) with previously established ID and newly diagnosed neurocognitive decline as the first manifestation of an IMD (10 per year). Of the LSD patients with ID and neurocognitive decline, 7.1% were referred to neuropsychology services, compared with 7.3% of patients with general metabolic disorders and ID or neurocognitive impairment. For referrals to neuropsychiatry, the proportion was similar at 11.9% for LSD patients and 8.8% for general metabolic IMD patients with ID or neurocognitive impairment.

For general metabolic patients, 24 patients were referred to neuropsychiatry and 20 were referred to neuropsychology.

For LSD patients, 10 patients were referred to neuropsychiatry and 6 were referred to neuropsychology for their assessment and intervention.

## 4. Discussion

Several reviews have been published in recent years concerning the metabolic causes of ID, mostly reflecting expert opinions and individual expertise in the field of IMD [20,21,22,23]. This study outlines the prevalence of ID among a large multi-ethnic cohort of adults with various IMDs. The results of the study are meaningful and have already led to a development of a referral pathway to a tertiary neuropsychiatry service, appointing a neuropsychologist and a mental health nurse to support our service.

In our cohort of 2268 patients, 1598 patients had general metabolic conditions (70.5%), and 670 had LSDs (29.5%). The pooled prevalence of ID and neurocognitive decline was found to be 15.7% (*n* = 357). In some cases, an individual presenting with an IMD plus ID or cognitive decline will also have other co-morbidities including epilepsy, cardiac abnormalities, or endocrine disorders [23]. Among our patients, neurological manifestations (epilepsy, leukodystrophy, spasticity, balance problems) were the most prevalent among patients with general metabolic disorders. Among patients with LSDs, apart from the neurological presentations, cardiopulmonary manifestations and skeletal abnormalities were common. The milder features are often present in patients in whom a late-onset IMD is considered in their adolescence or adulthood [20].

**Figure 3:**
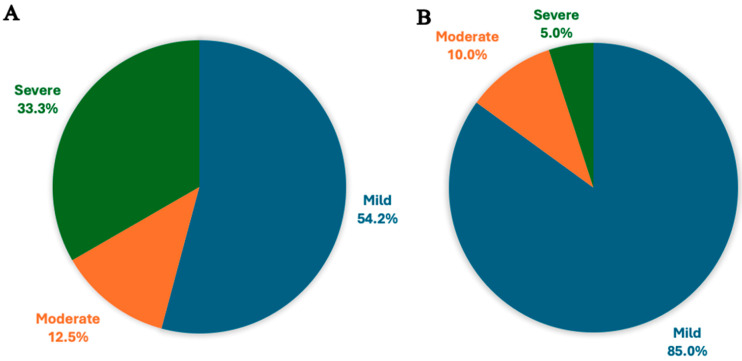
Referral patterns for patients with ID or neurocognitive impairment on a background of general metabolic disorders, by ID severity. (**A**) The number of patients with general metabolic ID who were referred to neuropsychiatry, categorised by the severity of their condition. The severity levels are divided into three groups: mild, moderate, and severe. There were 13 patients classified as having mild severity, 3 patients with moderate severity, and 8 patients with severe severity. (**B**) The number of patients with general metabolic ID who were referred to neuropsychology, categorised by the severity of their condition. The severity levels are divided into three groups: mild, moderate, and severe. There were 17 patients classified as having mild severity, 2 patients with moderate severity, and 1 patient with severe severity.

**Figure 4:**
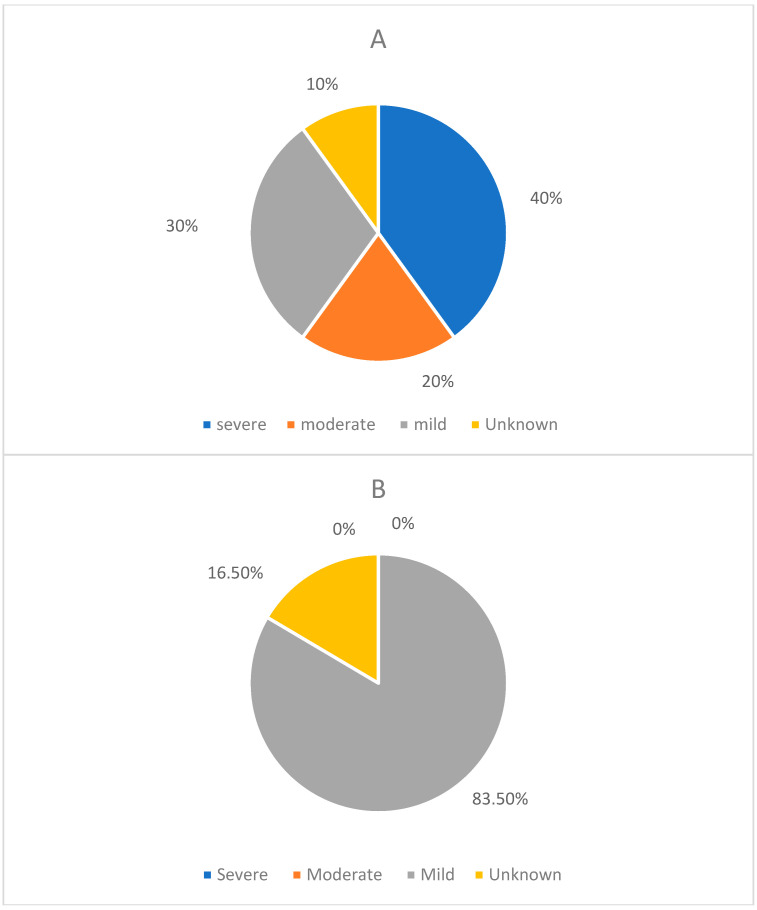
Referral patterns of patients with ID on a background of LSDs to neuropsychiatry and neuropsychology. (**A**) The number of patients referred to neuropsychiatry, categorised by the severity of their condition. The severity levels are divided into four groups: mild, moderate, severe, and not categorized. There were 3 patients classified as having mild severity, 2 patients with moderate severity, 4 patients with severe severity, and 1 patient with an unknown severity level. (**B**) The number of patients referred to neuropsychology, categorised by the severity of their condition. The severity levels are divided into two groups: mild and not categorized. There were 5 patients classified as having mild severity and 1 patient with an unknown severity level.

Over the 5 years up to 2023, we observed a twofold increase in referrals (Figure 3 and Figure 4) of adult metabolic patients with either ID or neurocognitive decline as the first manifestation of an IMD, highlighting the growing need for neuropsychology and neuropsychiatry services in metabolic medicine. In our clinic, supported decision making occurs with a trusted metabolic clinician and multidisciplinary team, who act in the best interest of the patient. Hence, any intervention and benefits of therapy are often discussed as part of best-interest meetings—with the intervention considered only if it is to improve the patient’s well-being and to treat a reversible and life-threatening abnormality. The referral pathways between tertiary metabolic, neuropsychiatry, and neuropsychology services allow for specialist assessments, intervention, and robust management. Many of our patients reside a considerable distance from our centre. Consequently, we conduct the initial IMD assessment at our facility, and subsequent follow-ups are arranged locally, close to their home address. The large catchment area poses a challenge for providing consistent long-term follow-up care.

Treatment choices for IMD patients with IMD/ID are often constrained by various factors that must be considered to enhance therapeutic adherence. Key factors include physical complications stemming from the disorder, the patient’s social support system, and the methods of drug administration. The interventions delivered included the neuropsychology assessment of cognitive, emotional, and behavioural problems and offering tailored support for these. The role of the neuropsychiatrist in these cases includes tertiary advice and guidance to psychiatrists regarding the diagnostic assessment of individuals with ID and possible IMD; the assessment and diagnosis of mental health problems in patients with ID and IMD, tackling diagnostic questions regarding parallel neurodegenerative disorders in patients presenting with cognitive decline; and specialist prescribing for mood and anxiety disorders, psychosis, sleep–wake disturbance, and agitation and aggression conferring risks. All the patients with limited capacity required individualised care formulated using best-interest meetings. In summary, neuropsychology and neuropsychiatry have an integral role in metabolic multidisciplinary teams, as evidenced by the results of this study. Several adults presented with neuropsychiatric symptoms (psychosis, behavioural problems, personality change, seizures, etc.) as the first clinical manifestation, and subsequent diagnostic work-up agreed upon by the metabolic and neurology/neuropsychiatry teams led to a definite IMD diagnosis. This highlights the role of neuropsychiatry in the diagnosis and further symptom management of these patients. One of the neurologists in our Trust completed a PhD thesis entitled “*The occurrence of metabolic and inflammatory causes of psychosis in admissions to a psychiatric unit*” in 2018, illustrating the joint MDT work that has taken place in our tertiary centre over the last decade.

The need for multiple tests to exclude a few rare to ultra-rare conditions and the limited availabilities of laboratories offering comprehensive diagnostic testing explains why outside highly specialised centres, metabolic work-ups of patients with ID may be incomplete in many cases, resulting in a potentially low diagnostic yield of metabolic testing. The diagnostic work-up includes white cell enzyme, PPCS biomarker, plasma amino acids, urine organic acids, the acylcarnitine profile, total homocysteine, plasma ammonia, plasma lactate, urine oligosaccharides, and glycosaminoglycans [24].

At our centre, notable cases of delayed diagnosis (*n* = 40) include a man with Tay-Sachs disease who had a 30-year history of depression and progressive decline in his neurological function before he was diagnosed with this condition. Another example is a 25-year-old female who presented with a seizure that led to further neurocognitive decline, and she has never recovered from it. She was diagnosed with SSADH and is institutionalised now, while previously she was fully independent. Also, cases at our centres exist regarding patients missed before newborn screening for metabolic conditions was introduced in 1969, who were then diagnosed with PKU in their 70s [25]. Also notable are cases of late-onset Niemann–Pick C disease, presenting with behavioural problems, slurred speech, and balance problems, before they develop memory problems and mobility issues leading to wheelchair dependence. Other cases include late-diagnosed α Mannosidosis, GM2 [26], Mucopolysaccharidosis type IIIA [27], MTHFR [28], MPS I [29], Mucolipidosis type III, Hawkinsuria, Cerebrotendinomatous xanthomatosis, or Congenital Glycosylation Defect Type I.

Concerning limitations, we acknowledge that this is a case series from only one centre. However, the IMD clinic is located in a tertiary centre for IMD, neuropsychiatry, and neuropsychology. Another limitation is the data categorisation and analysis. It was a retrospective data analysis, and patients were diagnosed by their paediatric team before the transition to the adult metabolic centre. The diagnosis of mild, moderate, and severe ID was often transcribed from historical records. To minimise human error, the selected patients’ diagnoses were proactively reviewed by our clinical team who care for these patients. There is a possibility that a diagnosis of mild ID was missed and never recorded, potentially leading to an underestimation of the number of patients in the mild ID group. Furthermore, we have not examined how the treatment of IMDs may mitigate the course or consequences of ID/neurocognitive decline. We recognise that the limitations impact the interpretation of the study results and constrain our ability to generalize from these results. The study is a platform for further research with prospective formal neuropsychology assessment, the application of available algorithms/software [13], and the development of guidelines for adults presenting with an IMD and ID.

Finally, multicentre research is needed to evaluate and further develop effective neuropsychological and neuropsychiatric interventions for this patient group.

## 5. Conclusions

In conclusion, neurocognitive and neuropsychiatric presentations can be the first presenting symptoms in late-onset IMDs. There is a clinical need for improved care, referral, and diagnostic pathways and training programmes across community, secondary, and tertiary teams, for patients with IMDs who have ID. Closer collaborative work between neuropsychiatry and metabolic teams and the joint patient review in clinics facilitates the diagnosis of IMDs in adult patients with ID and the commencement of treatment, which may positively impact their clinical outcomes.

## Figures and Tables

**Table 1:** Data on the prevalence of ID and neurocognitive impairment among patients with various general metabolic conditions. Includes the percentage of ID and cognitive impairment patients per condition and the percentage of patients with ID or neurocognitive impairment as a proportion of the total ID and neurocognitive impairment cohort. * Of those with homocystinuria, 3.2% (*n* = 2) had comorbid methylmalonic aciduria. ** Of those with methylmalonic aciduria, 14.3% (*n* = 2) had comorbid homocystinuria.

General Metabolic Condition	Percentage of ID/CI Patients per the Disorder in Our Cohort (Total Number with ID within Condition)	Percentage of Patients with ID/CI among the Whole Group with ID/CI
PKU	9.7 (38)	13.9
Classical galactosaemia	49.3 (34)	12.5
Undefined possible metabolic/genetic disorder	10.7 (17)	6.2
Homocystinuria *	17.5 (11)	4.0
GLUT 1 deficiency	55.6 (10)	3.7
Arginase deficiency	100.0 (8)	2.9
4 Hydroxybutyric Aciduria (Succinic Semialdehyde Dehydrogenase Deficiency)	100.0 (7)	2.6
OTC deficiency	22.6 (7)	2.6
Smith–Lemli–Opitz Syndrome	77.8 (7)	2.6
Cerebrotendinous xanthomatosis (CTX)	100.0 (7)	2.6
Methylmalonic Acidaemia **	42.8 (6)	2.2
MTHFR deficiency	50.0 (6)	2.2
Citrullinaemia	60.0 (6)	2.2
CDG 1a (Phosphomannomutase II PMM2 deficiency)	100.0 (5)	1.8
Argininosuccinic aciduria	62.5 (5)	1.8
MSUD	26.3 (5)	1.8
L2-Hydroxyglutaric aciduria	100.0 (4)	1.5
GAMT Deficiency	100.0 (4)	1.5
MCADD	5.6 (4)	1.5
McArdle’s disease	20.0 (4)	1.5
TANGO 2	80.0 (4)	1.5
Lowe’s syndrome	100.0 (3)	1.1
Propionic Acidaemia	60.0 (3)	1.1
SRD5A3-CDG	75.0 (3)	1.1
GTP Cyclohydrolase deficiency (6-Pyrovyltetrahydrobiopterin synthase Def)	100.0 (3)	1.1
Other	N/A (62)	22.7

**Table 2:** Data on the prevalence of ID and neurocognitive impairment among patients with (LSDs). Includes the percentage of ID/neurocognitive impairment (CI) patients per condition, and the percentage of patients with ID/neurocognitive impairment (CI) as a proportion of the total ID/neurocognitive impairment cohort.

LSD	Percentage of ID/CI Patients per Condition (Total Number with ID within Condition)	Percentage of Patients with ID/CI (% of ID/CI Cohort)
Niemann Pick C	100.0 (22)	26.2
MPS I	28.8 (13)	15.5
α-mannosidosis	100.0 (11)	13.1
MPS IIIa	100.0 (8)	9.5
MPS II	40.0 (6)	7.1
MPS IIIb	100.0 (5)	6.0
Tay-Sach’s Disease	100.0 (5)	6.0
MPS VI	12.5 (4)	4.8
Mucolipidosis IV	100.0 (2)	2.4
β Mannosidosis	100.0 (2)	2.4
Aspartylglucosaminuria	100.0 (2)	2.4
MPS IIIc	33.3 (1)	1.2
Battens-CLN2	100.0 (1)	1.2
MPS VII	10.0 (1)	1.2
Schindler’s disease	100.0 (1)	1.2

## Data Availability

The data presented in this study are available on request from the corresponding author.
